# Sex-specific regional grey matter volume correlates of daily activities

**DOI:** 10.1038/s41598-018-28252-w

**Published:** 2018-07-02

**Authors:** Tsukasa Ueno, Naoya Oishi, Toshiya Murai

**Affiliations:** 1 Department of Psychiatry, Kyoto University Graduate School of Medicine, Kyoto, Japan; 2 Research and Educational Unit of Leaders for Integrated Medical System, Center for the Promotion of Interdisciplinary Education and Research, Kyoto University, Kyoto, Japan

## Abstract

The human brain is plastic and continuously modified throughout life by our daily experiences and behaviours. However, no reports have comprehensively investigated structural brain correlates of our daily activities, including possible sex differences. In this study, we examined the relationship between a self-reported 24-hour Life-Log and regional brain volume measured by structural magnetic resonance imaging. We analysed brain volumes of 64 males and 53 females that were obtained from multiple scanning sites. We found several sex-specific correlations, including a positive correlation between superior frontal gyrus (Brodmann area 8) volume and domestic work hours, and a negative correlation between volume in the same region and job-work hours. Despite being a cross-sectional study, this study provides empirical evidence for how and to what extent brain structure is correlated with everyday activity.

## Introduction

The brain is plastic and continuously modified throughout life by our daily experiences and behaviours. Structural brain imaging techniques, particularly magnetic resonance imaging (MRI), allows visualization of experience/behaviour-dependent plastic changes in the human brain. This topic was explored in Maguire and colleague’s seminal study that showed how the posterior hippocampal volume of London taxi drivers was correlated with their taxi-driving experience^1^. Practice/training-dependent cortical plasticity has also been demonstrated in the auditory cortex of professional musicians and in the visual motion area of beginning jugglers^2,3^.

In contrast to these early studies that investigated the effect of specific practices on specific brain structures, recent studies have focused on more general daily habits or lifestyles. For example, recent studies investigating middle-aged and older adults have shown that hippocampal volume was positively correlated with time spent exercising^4,5^.

Aside from exercise, other regular daily activities include social activity and sleeping. In one longitudinal study, low-quality sleep in older people was shown to be associated with smaller volume within the superior frontal cortex and a greater atrophy rate across the frontal, temporal, and parietal cortices^6^. In studies related to social interaction, the size and complexity of social networks was shown to be correlated with amygdala volume, and the size of online social networks was shown to be correlated with volume of the left middle temporal and right superior temporal gyri, as well as the right entorhinal cortex^7,8^.

In addition to the amount or length of each activity, activity regularity might also influence plastic changes in the brain. Irregular lifestyles are thought to have a negative effect on cognition and the brain in general. Indeed, a prospective cohort study of employed workers demonstrated that shift work for more than 10 years impaired cognitive function in several domains^9^. Furthermore, extended shift working seems to affect brain structure, as evidenced by the lower cognitive performance and smaller right temporal lobe volume in cabin crews who experienced chronic exposure to difficult shift work, with jet-lag recovery periods that were less than 5 days^10^.

Thus, studies indicate that structural changes in the brain can be associated with the amount and regularity of particular daily activities. However, to date, each study has focused on a specific type of daily habit or activity, and no reports have comprehensively investigated the structural brain correlates of a 24-hour schedule of daily activities.

Here, we aimed to examine the relationships between regional brain volumes in healthy adults and their hours spent and weekly variation of a number of daily activities. To assess daily activities and regional brain volumes comprehensively, we use a self-reported 24-hour Life-Log in conjunction with structural MRI from multiple centres. Males and females were investigated separately because sex differences in brain structures are known to be substantial; across all ages, total brain volume is consistently reported to be about 10% larger in males than in females. Additionally, regional differences in the cerebral cortex related to sex have also been reported^11,12^. Furthermore, our preliminary analysis of the Life-Log showed substantial sex differences in lifestyle, which further justifies analysing each sex’s data separately.

## Results

### Demographic and 24-hour Life-Log data

One hundred and seventeen participants (64 males and 53 females) were included in the final analysis after meeting the inclusion criteria. Table 1 shows their physical characteristics information. The physical characteristics for all 144 participants can be seen in Supplementary Table S1. No demographic variables differed significantly between the males and females. The 24-hour Life-Log data indicated that male participants spent significantly more time engaged in Commuting to Work/School, Job-work, Sports, and Being with Classmates/Colleague compared with females. Conversely, female participants spent significantly more time engaged in Domestic-work, Caring/Nursing, and Shopping. As for the weekly range, male participants had a significantly higher range for Commuting to Work/School, Job-work, Rest/Relaxation, Hobbies/Amusement, Being with Family, and Being with Classmate/Colleagues, and female participants had a higher range for Domestic-work and Non-commute Travel (Tables 2 and 3). The descriptive statistics of all participants are shown in Supplementary Table S2.

**Table 1:** Physical characteristics of participants.

	Male (n = 64)			Female (n = 53)		
	Mean	SD	Range	Mean	SD	Range
Age (years)	49.9	8.8	25–69	47.0	7.6	32–68
Body Mass Index (BMI)	22.9	3.0	17.5–31.9	21.0	3.4	14.6–34.1
Systolic Blood Pressure (mmHg)	128.9	14.2	100–159	120.8	18.3	83–167
Diastolic Blood Pressure (mmHg)	83.3	10.0	63–110	74.2	12.2	46–105
Heart Rate (per minute)	70.3	11.2	49–101	69.7	10.2	49–101

**Table 2:** Average daily activity.

Average daily activities (hours)	Male (n = 64)				Female (n = 53)				Statistics
	Mean	SD	Range	n (for VBM)	Mean	SD	Range	n (for VBM)	P-value
Sleep	6.7	0.9	4.6–9.0	64	6.8	0.9	4.7–8.7	53	0.48^a^
Personal Care	1.1	0.6	0.0–3.2	63	1.1	0.7	0.0–3.3	53	0.94^a^
Meals	1.7	0.6	0.4–3.7	63	1.8	0.6	0.6–3.2	53	0.65^a^
Commuting to Work/School	1.5	0.6	0.0–3.3	64	0.8	1.1	0.0–7.6	52	<10^−6 b ***^
Job-work	6.8	1.7	0.0–10.9	63	3.5	3.0	0.0–0.9	53	<10^−7 b ***^
School-work	0.0	0.0	0.0–0.2	n/a	0.0	0.3	0.0–2.5	52	0.10^b^
Domestic-work	0.2	0.5	0.0–2.8	63	3.2	2.6	0.0–11.4	51	<10^−12 b ***^
Caring/Nursing	0.0	0.0	0.0–0.2	n/a	0.1	0.3	0.0–1.7	52	0.15^b^
Child Care	0.2	0.7	0.0–3.5	62	0.1	0.6	0.0–3.3	51	0.51^b^
Shopping	0.2	0.2	0.0–1.0	n/a	0.7	0.6	0.0–2.7	52	<10^−6 b ***^
Non-commute Travel	0.3	0.7	0.0–5.2	63	0.4	0.7	0.0–4.0	52	0.01^b^
Television/Radio/Newspaper/Magazine	1.5	1.2	0.0–5.0	64	1.8	1.6	0.0–6.5	53	0.48^b^
Rest/Relaxation	1.6	1.0	0.0–4.4	64	1.6	1.3	0.0–5.8	52	0.80^b^
Learning/Self-development	0.1	0.5	0.0–2.5	61	0.1	0.2	0.0–1.3	51	0.87^b^
Hobbies/Amusements	0.6	0.8	0.0–3.8	63	0.5	1.0	0.0–6.7	52	0.05^b^
Sports	0.3	0.8	0.0–6.4	63	0.2	0.5	0.0–3.3	52	0.01^b*^
Volunteer/Community Activity	0.0	0.3	0.0–2.5	63	0.0	0.4	0.0–2.2	51	0.90^b^
Other Social Activities	0.3	0.8	0.0–3.7	62	0.2	0.5	0.0–2.1	51	0.97^b^
Hospital Visit/Treatment	0.0	0.0	0.0–0.0	n/a	0.0	0.0	0.0–0.5	n/a	0.27^b^
Other Activities	0.0	0.1	0.0–0.9	61	0.1	0.5	0.0–3.0	51	0.68^b^
Being Alone	8.0	5.6	0.0–22.2	64	7.4	5.5	0.0–19.6	53	0.55^b^
Being with Family	7.5	5.3	0.0–19.2	64	9.8	6.7	0.0–24.0	53	0.06^b^
Being with Classmates/Colleagues	6.7	2.8	0.0–10.7	64	3.5	3.3	0.0–11.2	53	<10^−5 b ***^
Being with Other People	0.8	1.6	0.0–7.2	62	0.5	0.8	0.0–2.9	53	0.64^b^

^*^p < 0.05, ^**^p < 0.01, ^***^p < 0.001, ^a^t-test, ^b^Mann-Whitney U test, n/a: not applicable.

**Table 3:** Average weekly range of activity.

Average weekly range (hours)	Male (n = 64)				Female (n = 53)				Statistics
	Mean	SD	Range	n (for VBM)	Mean	SD	Range	n (for VBM)	P-value
Sleep	1.5	1.2	0.0–6.0	63	1.2	1.1	0.0–6.7	52	0.08^b^
Personal Care	0.8	0.9	0.0–4.5	62	0.7	0.7	0.0–2.5	53	0.48^b^
Meals	0.7	0.6	0.0–3.0	63	0.6	0.6	0.0–2.5	53	0.30^b^
Commuting to Work/School	2.1	0.9	0.0–4.0	64	1.2	1.6	0.0–10.7	51	<10^−6 b***^
Job-work	9.3	2.1	0.0–14.0	63	5.0	4.1	0.0–11.2	53	<10^−7 b ***^
School-work	0.0	0.1	0.0–1.0	n/a	0.1	0.5	0.0–3.5	52	0.11^b^
Domestic-work	0.6	0.9	0.0–3.5	64	1.8	1.4	0.0–7.0	52	<10^−6b ***^
Caring/Nursing	0.0	0.1	0.0–0.7	n/a	0.1	0.5	0.0–2.5	51	0.16^b^
Child Care	0.5	1.4	0.0–7.5	62	0.2	0.8	0.0–5.0	52	0.30^b^
Shopping	0.8	0.9	0.0–3.0	64	1.1	1.2	0.0–6.5	52	0.15^b^
Non-commute Travel	0.6	1.1	0.0–6.2	63	0.7	1.1	0.0–5.0	51	0.04^b*^
Television/Radio/Newspaper/Magazine	2.0	1.9	0.0–8.5	63	1.8	1.9	0.0–8.2	52	0.56^b^
Rest/Relaxation	2.2	1.6	0.0–6.5	64	1.4	1.2	0.0–4.0	53	0.01^b*^
Learning/Self-development	0.2	0.8	0.0–5.2	62	0.3	0.9	0.0–4.7	51	0.98^b^
Hobbies/Amusements	1.8	2.2	0.0–9.0	63	0.8	1.6	0.0–6.5	51	0.007^b **^
Sports	1.1	1.9	0.0–10.7	63	0.4	1.0	0.0–5.7	51	0.002^b **^
Volunteer/Community Activity	0.1	0.5	0.0–3.5	62	0.2	1.4	0.0–9.7	52	0.93^b^
Other Social Activities	0.5	1.6	0.0–11.5	63	0.9	2.0	0.0–10.0	51	0.62^b^
Hospital Visit/Treatment	0.0	0.0	0.0–0.0	n/a	0.0	0.1	0.0–0.7	n/a	0.27^b^
Other Activities	0.1	0.6	0.0–4.5	63	0.1	0.7	0.0–4.0	50	0.43^b^
Being Alone	4.0	3.4	0.0–12.5	64	4.7	4.3	0.0–19.2	52	0.64^b^
Being with Family	6.9	5.0	0.0–17.0	64	4.7	4.3	0.0–18.0	52	0.01^b*^
Being with Classmates/Colleagues	9.4	3.8	0.0–15.0	64	5.0	4.6	0.0–13.5	53	<10^−5b ***^
Being with Other People	1.6	2.9	0.0–13.0	62	1.5	2.5	0.0–11.0	51	0.92^b^

^*^p < 0.05, ^**^p < 0.01, ^***^p < 0.001, ^a^t-test, ^b^Mann-Whitney U test, n/a: not applicable.

### Main activities

**Figure 1:**
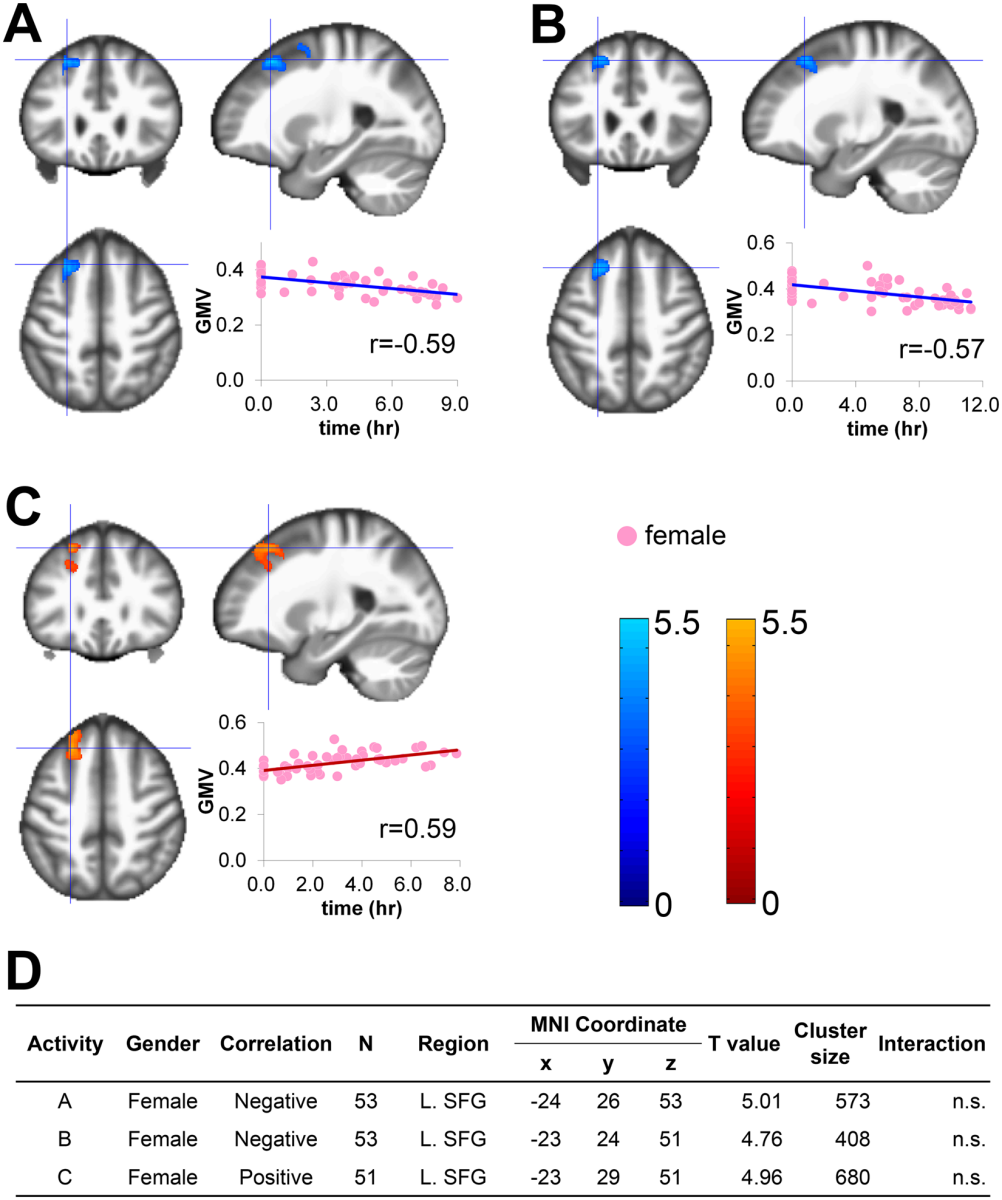
Sex-specific correlations between grey matter volume (GMV) and each item in main activities. Regions that survived a statistical threshold of p < 0.05 (cluster-level corrected for multiple comparisons) are shown with (**A**) daily time of Job-work in females, (**B**) weekly range of daily time of Job-work in females, and (**C**) daily time of Domestic work in females (**D**) Summary of the significant correlations. The X-axis of the scatter plot indicates the daily time or weekly range of activities. The Y-axis indicates the extracted GMV over the peak coordinates of the significant cluster. L, left; MNI, Montreal Neurological Institute; SFG, superior frontal gyrus; n.s.: not significant.

In females, the daily time and weekly range for Job-work were significantly negatively correlated with grey matter volume (GMV) in the left superior frontal gyrus (coordinates for Job-work hours: x = −24, y = 26, z = 53, r = −0.59, cluster size 573 voxels; Job-work weekly range: x = −23, y = 24, z = 51, r = −0.57, cluster size 408 voxels; Fig. 1A,B), and daily time for Domestic-work was significantly positively correlated with GMV in the left superior frontal gyrus (x = −23, y = 29, z = 51, r = 0.59, cluster size 680 voxels; Fig. 1C). In contrast, males did not exhibit any significant correlations between regional GMV and daily hours or weekly range for any of the main activities. Sex by activity interactions with respect to GMV were not significant for daily Job-work time, the weekly range of Job-work, or daily Domestic-work time (Fig. 1).

### Secondary activities

**Figure 2:**
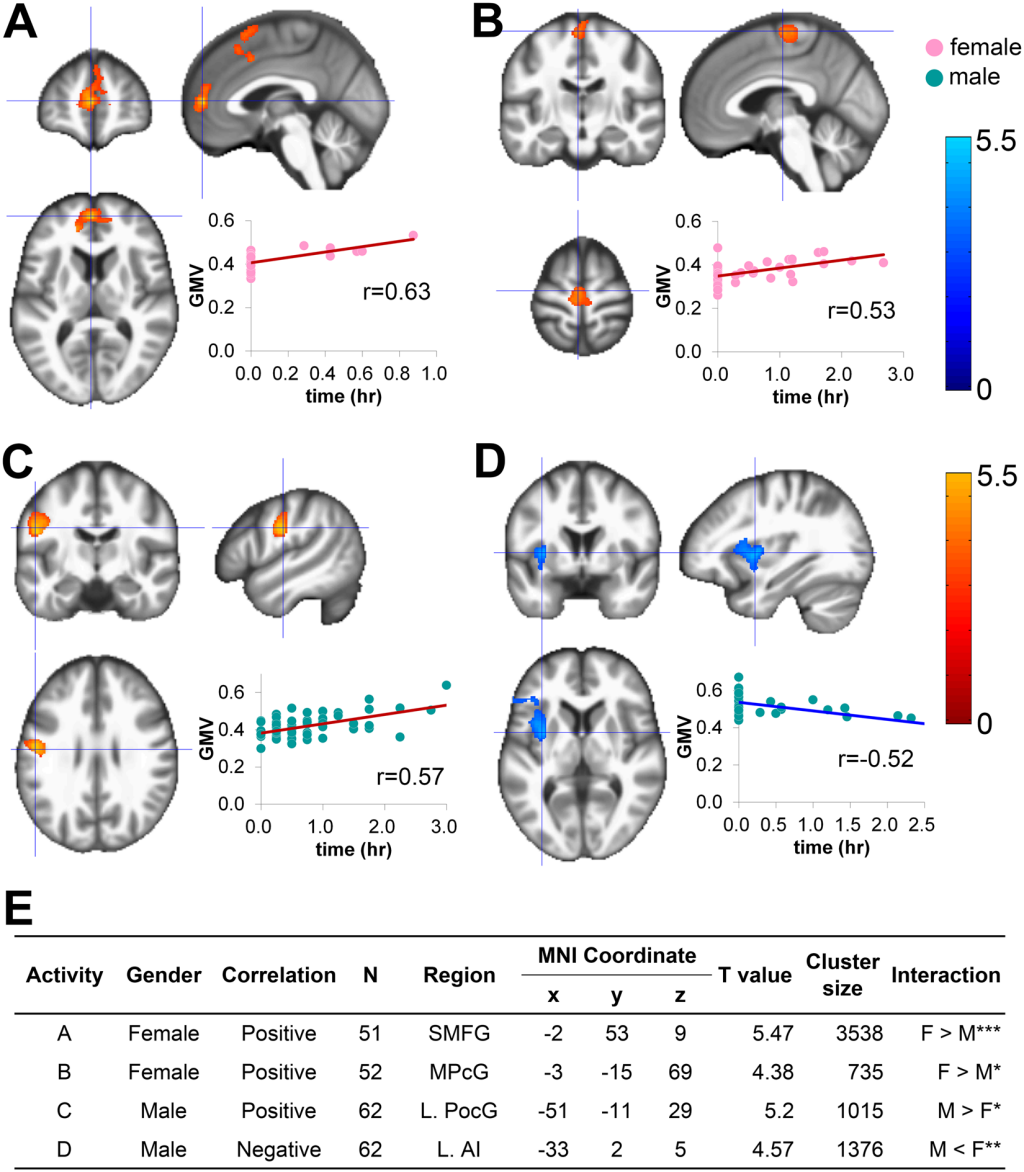
Sex-specific correlations between GMV and each secondary activity item. Regions that survived a statistical threshold of p < 0.05 (cluster-level corrected for multiple comparisons) are shown with (**A**) daily time of Learning/self-development in females, (**B**) daily time of Hobbies/amusements in females, (**C**) weekly range of daily time of Personal care in males, and (**D**) daily time of Other social activities in males. (**E**) Summary of the significant correlations. The X-axis of the scatter plot indicates the daily time or weekly range of activities. The Y-axis indicates the extracted GMV over the peak coordinates of the significant cluster. L, left; MNI, Montreal Neurological Institute; AI, anterior insula; PocG, postcentral gyrus; MPcG, medial precentral gyrus; SMFG, superior medial frontal gyrus. ^*^p < 0.05, ^**^p < 0.01, ^***^p < 0.001.

In females, daily hours for Learning/Self-development were significantly and positively correlated with GMV in the superior medial frontal gyrus (r = 0.63; Fig. 2A), and that for Hobbies/Amusements was significantly and positively correlated with GMV in the medial precentral gyrus (r = 0.53; Fig. 2B). In males, the weekly range for Personal Care was significantly and positively correlated with GMV in the left postcentral gyrus (r = 0.57; Fig. 2C) and daily time for Other Social Activities was significantly and negatively correlated with GMV in the left anterior insula (r = −0.52; Fig. 2D). Sex by activity interactions in terms of GMV were significant for daily time spent engaged in Leaning/Self-development, daily time spent engaged in Hobbies/Amusements, the weekly range of Personal Care, and daily time spent Being with Other People (Fig. 2).

### Sleep, rest, and relaxation

**Figure 3:**
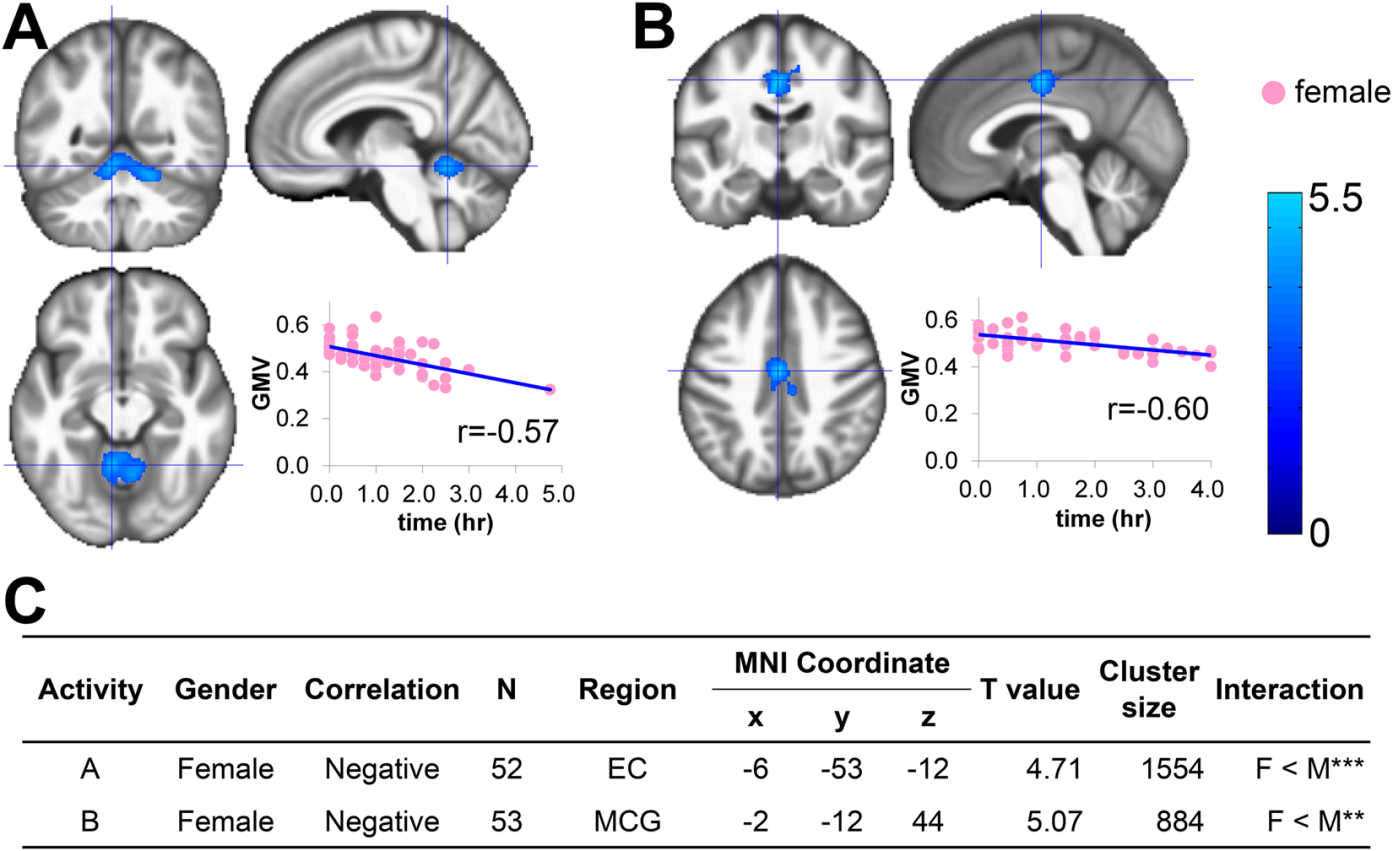
Sex-specific correlations between GMV and hours of sleep, rest, and relaxation. Regions that survived a statistical threshold of p < 0.05 (cluster-level corrected for multiple comparisons) are shown with (**A**) weekly range of daily time of Sleep in females, and (**B**) weekly range of daily time of Rest/relaxation in females. (**C**) Summary of the significant correlations. The X-axis of the scatter plot indicates the daily time or weekly range of activities. The Y-axis indicates the extracted GMV over the peak coordinates of the significant cluster. MNI, Montreal Neurological Institute; EC, exterior cerebellum; MCG, middle cingulate gyrus ^*^p < 0.05, ^**^p < 0.01, ^***^p < 0.001.

In females, the weekly range for Sleep was significantly and negatively correlated with GMV in the exterior cerebellum (r = −0.57; Fig. 3A) and that for Rest/Relaxation was significantly and negatively correlated with GMV in the middle cingulate gyrus (r = −0.60; Fig. 3B). In contrast, males exhibited no significant correlations between regional GMV and daily time or weekly range for any of the activities within this category. Sex by activity interactions in terms of GMV were significant for the weekly range of Sleep and the weekly range of Rest/Relaxation (Fig. 3).

### Social and interpersonal activities

**Figure 4:**
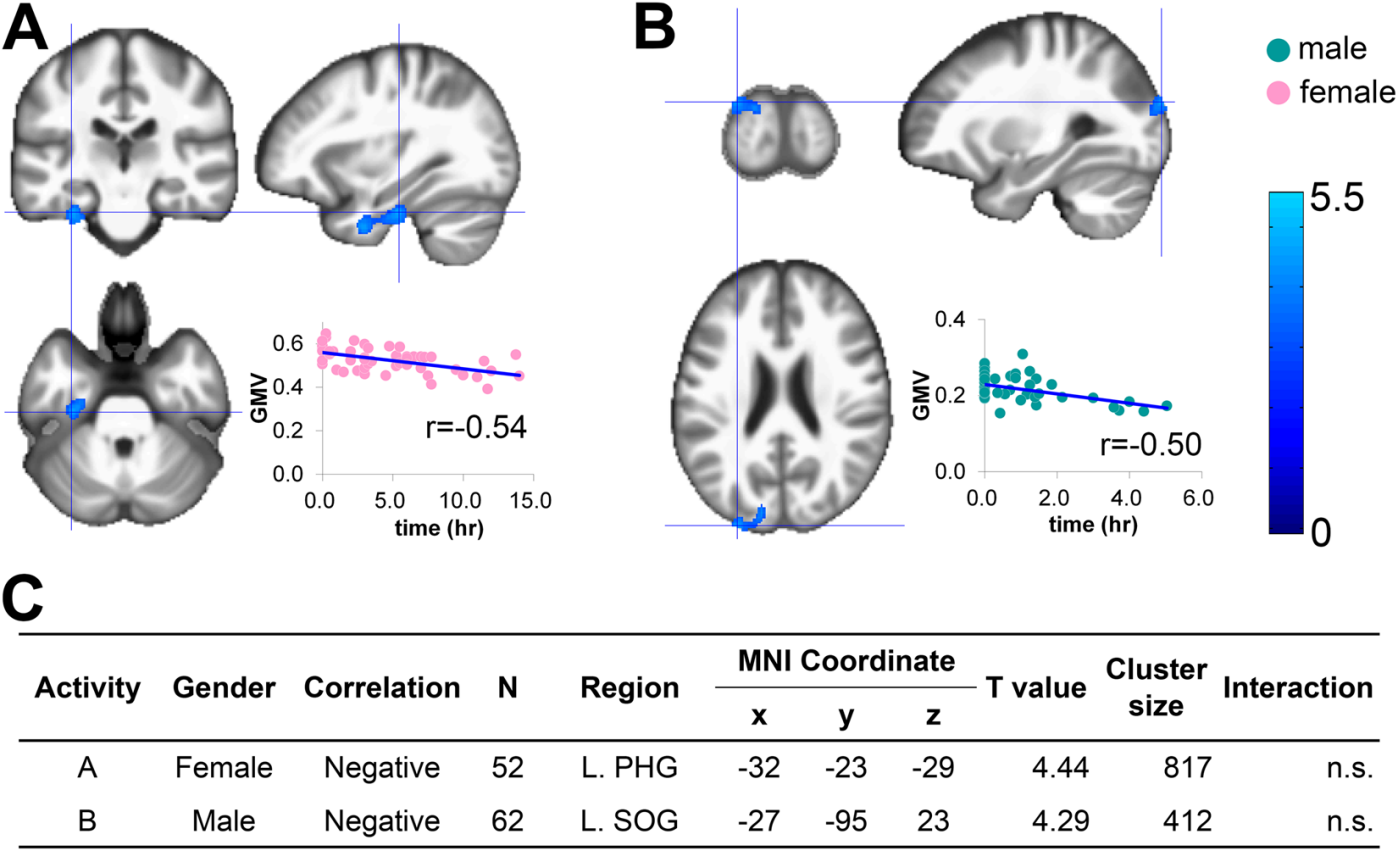
Sex-specific correlations between GMV and each item from the social/interpersonal activities. Regions that survived a statistical threshold of p < 0.05 (cluster-level corrected for multiple comparisons) are shown in (**A**) weekly range of daily time being with Family in females, and (**B**) daily time of being with Other persons in males. (**C**) Summary of the significant correlations. The X-axis of the scatter plot indicates the daily time or weekly range of activities. The Y-axis indicates the extracted GMV over the peak coordinates of the significant cluster. L, left; MNI, Montreal Neurological Institute; PHG, parahippocampal gyrus; SOG, superior occipital gyrus; n.s.: not significant.

In females, the weekly range for time spent Being with Family was significantly and negatively correlated with GMV in the left parahippocampal gyrus (r = −0.54; Fig. 4A). In males, daily time for Being with Other People was significantly and negatively correlated with GMV in the left superior occipital gyrus (r = −0.50; Fig. 4B). Sex by activity interactions in terms of GMV were not significant for the weekly range of Being with Family and the daily time spent Being with Others (Fig. 4).

## Discussion

This study revealed that regional brain volumes were related not only to primary activities such as working but also with those such as hobbies and relaxation. All of the significant correlations were found to be sex-specific. Although previous studies have shown associations between specific training/practice and specific structural changes in the brain, no study has comprehensively examined how normal daily activities performed by all people affect the regional structure of the brain. Compared with intensive training, such as juggling or specific cognitive training adopted by patients with neuropsychiatric problems, the effect on the brain of a single day of normal daily activities might be small^3,5,13^. However, with repetition over extended periods, these kinds of daily activities might have a greater potential for promoting plastic change in the brain than do planned intensive interventions.

Regarding the primary activities, we found an “inverse law” related to Job-work and Domestic-work in females. The lateral surface of the dorsal part of the left superior frontal gyrus (Brodmann area 8, Fig. 1A,B) was smaller for females who reported longer Job-work hours or larger weekly ranges. In contrast, the same area was larger in females who reported longer Domestic-work hours (Fig. 1C). This region, situated within the left dorsolateral prefrontal cortex, is involved in higher cognitive functions, particularly working memory, spatial processing, and attention related to visual perception^14,15^. Working memory is a fundamental cognitive process for keeping things in mind while performing complex tasks such as reasoning and learning, and is essential for performance of our day-to-day activities^16^. Although no study has directly discussed the association of domestic work with cognitive function, a longitudinal survey comparing databases from 1974 with those from 1994 reported that complication of domestic work in modern society led to increases in intellectual flexibility^17^. Although this topic has not yet gained much attention, complexity of domestic work might recruit more working memory, and thus promote plastic change in the related brain regions. Interestingly, volumes of the same brain region were inversely correlated with Job-work hours, again only in females. The female-only of these associations is interesting in the context of the social debate regarding fair sharing of domestic work. In Japan, females spend much more time doing domestic work than males, and domestic work is less valued than paid-work (2011 Survey, Summary of Results (Statistics Bureau, Ministry of Internal Affairs and Communications, http://www.stat.go.jp/english/data/shakai/2011/pdf/timeuse-a.pdf). Our results are somewhat surprising in that they demonstrate a positive impact of domestic work on our brain. Although preliminary, these findings might add another perspective on this socially hot debate. With respect to the inverse correlation between job-work activity and GMV in the same brain region, although it is possible that job-work activity has some unknown negative impact on the brain, it is more reasonable to explain this association from the viewpoint of a trade-off relationship between job-work and domestic-work activity. Indeed, in our female participants, domestic-work time was inversely correlated with job-work time (r = −0.702, p < 10^−8^). Thus, in our view, these two correlations should be interpreted as a set, instead of separately. Indeed, the association between domestic-work and GMV may reflect real causation, while the association between job-work and GMV may reflect the consequence of this relationship. Specifically, females who spent more time engaged in job-work spent less time engaged in domestic-work, and so may have been less likely to exhibit the increased GMV associated with domestic work.

We also found several sex-specific associations in the secondary activities. First, we demonstrated a female-specific correlation between time spent for Learning/Self-development and the volume of superior medial frontal gyrus (Fig. 2A). A cohort study indicated that participating in one or more pastime activities such as handcrafts, playing musical instruments, playing card games or board games, and reading was associated with less decline in cognitive and executive functions in older people^18^. The superior medial frontal gyrus is known to be a key region responsible for rapid inhibitory control, and inhibitory control of appropriate actions is an important aspect of executive function^19,20^. In our study, details were unavailable regarding the contents of the self-development or learning in which the participants were engaged. However, we speculate that they likely included highly demanding cognitive activities like reading and that high levels of executive function recruitment day after day promoted gradual plastic changes in the cortical areas responsible for these functions.

Another female-specific finding in the secondary activities was the positive correlation between Hobbies/Amusements with medial precentral gyrus volume (Fig. 2B). The precentral gyrus contains the primary motor cortex that controls voluntary movements of skeletal muscles. However, it is also known as a part of the default mode network, which is active when a person is mind-wandering and not focused on the outside world, and consists of bilateral and symmetrical cortical areas in the medial and lateral parietal, medial prefrontal, and medial and lateral temporal cortices^21,22^. Some studies have shown the effect of meditation on the default mode network, among which Creswell et. l reported that a 3-day intervention of mindfulness meditation training increased the functional connectivity in the default mode network^23–25^. The effect of concentrating on hobbies or amusements might be similar to that of meditation in the sense that both foster process-oriented approaches to the self.

We also demonstrated male-specific correlations in the data for secondary activities. The weekly range for Personal Care was positively correlated with the volume of the left postcentral gyrus (Fig. 2C). The postcentral gyrus, also known as the primary sensory cortex, is known to be activated when distinguishing the self from other^26,27^. Personal care such as face-washing, changing of clothes, or taking a shower might involve cognitive processes related to perspective-taking in relation to social emotions, and would thus be related to this brain region. In addition to Personal Care, time spent on Other Social Activities was negatively correlated with the volume of the left anterior insula (Fig. 2D). The anterior insula is a multifunctional region involved in various cognitive, perceptual, and socio-emotional processes^28^. The literature does not conclusively indicate whether the quantity of social activities has a positive or negative effect on our cognition/brain. For example, while one study has reported that social activities ameliorated deterioration of cognitive functions with aging and reduced the risk of depression in middle age, another has reported that negative social interaction was a risk factor for mild cognitive impairment in old age^29,30^. Our results might reflect the second case; an adverse association through the shrinkage of the anterior insula, which is a brain region where social and emotional processing intersect. Alternatively, this inverse correlation might be similar to the inverse correlation we observed between Job-work time and GMV in females. Given the trade-off relationship among activities, longer time spent engaged in social activities necessarily means shorter time engaged in other activities. Thus, the demonstrated inverse correlation might be the consequence of another relationship, although in this case we were not able to identify a possible causal relationship for which this correlation might be the consequence.

In females, the weekly range of sleeping time was negatively correlated with exterior cerebellum volume, while that for rest and relaxing was negatively associated with the middle cingulate gyrus (Fig. 3A,B). The finding for sleep might be related to the biological sleep-wake cycle that is under the control of a circadian oscillator that is reported to be located in the cerebellum^31,32^. The findings for Resting/Relaxation could be related to the roles of the middle and anterior cingulate cortices in self-regulation^33^. In line with this possibility, previous volumetric studies have shown that the volume of the middle cingulate cortex (together with the anterior cingulate cortex) is associated with meditation practice^34^.

Finally, in females, the weekly range for Being with Family was negatively correlated with the volume of the left parahippocampal gyrus (Fig. 4A), while in males, daily time for Being with Other People was negatively correlated with the volume of the left superior occipital gyrus (Fig. 4B). Both the parahippocampal and superior occipital gyri are known to be involved in the pathophysiology of anxiety and depressive disorders^35,36^. Thus, patterns of specific interpersonal relationships, which are likely to be related to the quantity and quality of mental stress, might be associated with the volume of these regions. However, the chance discoveries of these associations should be re-investigated in future studies.

Among the 11 significant correlations between activity measures (daily time or weekly range) and regional GMV among the participants, the results of the interaction analyses were also significant for some associations, but not others. For the former, we may make stronger claims as to the sex-specificity of the effect, while we can only make weak claims for the latter associations. As the association between domestic-work/job-work and regional GMV in females (Fig. 1) is one of the main findings of our study, we examined the reasons for the absence of significant interactions. As shown in Tables 2 and 3, the SDs of daily time spent engaged in domestic-work as well as job-work were very different between the male and female participants. This inhomogeneity of variance limits the appropriate interpretations of the results of the interaction analyses. The small SDs found for the male participants indicate that, compared with the female participants, males represented a much more homogeneous group in terms of the time spent engaged in these activities. The small inter-subject variability in time spent engaged in domestic-work and job-work in the male participants poses a challenge in terms of identifying the GMV correlates of these activities in males. The observed sex-differences in time spent engaged in domestic-work as well as job-work is likely explained by the gender-based differences in employment rate in Japanese society. Thus, future studies with more heterogeneous male samples in terms of employment status are necessary.

Our data contained a large number of zero value points, which could affect interpretation of relationships between activity and brain volume. To address this, we conducted group comparison analyses between zero value and a non-zero value groups, specifically, for daily hours spent engaged in Learning/Self-development in females, and daily hours spent being with Other People in males, because these activity variables included a large number of zeros (more than 40%; 45, 50, 33, 37 zeros, respectively). As results, group comparisons for these four significant correlations survived a statistical threshold of p < 0.05 (cluster-level corrected for multiple comparisons, p = 0.002, 0.04, 0.01, and 0.01, respectively). As results, group comparisons for these four significant correlations survived a statistical threshold of p < 0.05 (cluster-level corrected for multiple comparisons, p = 0.002, 0.04, 0.01, and 0.01, respectively).

This study has several limitations. First, the 24-hour Life-Log data of daily activities might not be sufficiently reliable as they are self-reported records, and the test-retest reliability of this log has not been examined thus far. In addition, we assessed only two representative days to evaluate the activity of the subjects, one “high activity day” and one “low activity day”. This is obviously an oversimplification of the variable daily activity of a person. Prolonged real-life monitoring would have been optimal. Although the Life-Log required participants to report a single representative activity for each time unit, individuals often perform multiple daily activities within the same period. Further, we did not collect data regarding social background or educational history. Educational history or IQ would likely be associated with employment rate, job category, and working hours, and these factors could influence the results. Furthermore, interaction among multiple activity variables could have influenced our results. For instance, the amount of time for one daily activity can affect that for others, such as the association of longer work with shorter sleep. As for statistical analyses, we tested a large number (176) of statistical models. Thus, our data may be affected by a multiple comparison problem. Further, due to the exploratory nature of our study, some of our discussion points were quite speculative. Further research is needed to address our preliminary findings. Finally, and most importantly, the cross-sectional design of our study cannot answer whether daily behaviour causes changes in brain volume or is the result of them.

In conclusion, we have demonstrated several sex-specific relationships between daily activities and regional GMV in healthy adults. The activities that were found to be associated with regional brain volume include primary components of our daily life, such as sleep and social interactions. Additionally, we also demonstrated intriguing correlations between other components of our daily lives, such as domestic work, hobbies, self-learning, and job work. Very little is known about whether these activities have positive or negative effects on our cognition, and nothing is known regarding the long-term effects on our brains. Our comprehensive approach using the Life-Log unearthed these associations. In the future, objective daily-activity monitoring using wearable devices, in conjunction with a longitudinal study design, would confirm or disconfirm our preliminary findings.

## Methods

### Participants

One hundred and forty-four volunteers (80 males and 64 females) were recruited in local cities in Hyogo (RIKEN), Kyoto (Kyoto University), and Tokyo (University of Tokyo), Japan^37^. All were Japanese adults (males: aged 46.9 ± 7.8 years; range, 25–69 years; females: 49.7 ± 8.1 years; 32–68 years). Participants were pre-screened to exclude any history of a psychiatric disorder, severe medical or neurological illness, or moderate to severe head injury. The population characteristics of participants are shown in Supplementary Table S1.

This study was approved by the ethics committees of RIKEN, Kyoto University, and the University of Tokyo and was performed in accordance with the guidelines and regulations of these research institutions^37^. All participants gave written informed consent before participation, and participant anonymity has been preserved.

### “24-hour Life-Log” and procedure

We used the self-reported “24-hour Life-Log” questionnaire in this study (Supplementary Figures 1, 2). All participants were instructed to assume a normal day within the past year, and complete the Log on the same day of MRI acquisition. We instructed participants to fill out the Life-Log for two type of days: typical “High Activity” day, which we expected to correspond to a regular weekday, and a typical “Low Activity” day, which we expected to correspond to regular weekend day. They were asked to indicate how many days in a week (7-day period) were typically High Activity and how many were Low Activity (for a total of seven). The 24 hours were divided into 15-minute blocks, and participants were required to fill out each block with one representative activity, even if they were engaged in two or more activities at the same time.

The 24-hour Life-Log documented an entire day’s activities and comprised a part of “questionnaire A” from the survey administered every 5 years by the Statistics Bureau of the Ministry of Internal Affairs and Communications (http://www.stat.go.jp/english/data/shakai/index.htm). The items were categorized into the following four types: (1) Primary activities (Meals, Commuting to Work/School, Job-work, School-work, Domestic-work, Caring/Nursing, Child Care, and Shopping); (2) Secondary activities (Non-commute Travel, Television/Radio/Newspaper/Magazine, Learning/Self-development, Hobbies/Amusements, Sports, Volunteer/Community Activities, Other Social Activities, Hospital Visit/Treatment, and Other Activities); (3) Sleep/Rest and Relaxation; (4) Social/Interpersonal Activities (Being Alone, Being with Family, Being with Classmates/Colleagues, and Being with Other People).

All 144 participants filled out the 24-hour Life-Log. Sixteen males and eleven females were excluded because they did not fill out the correct number of High or Low Activity days. In the remaining 117 participants, 11 males and 13 females recorded partial, overlapping, or deficient log entries. However, because the error rates were under 20% for each log, we included all the logs in the analysis.

From these Life-Logs, we calculated the “daily time” and “weekly range” for each activity. Daily time was calculated as the mean number of hours/day for a given activity (i.e., the total hours divided by the sum of High and Low Activity days). The “weekly range” of each activity was the absolute value of the difference between the mean hours for that activity on High and Low Activity days. For each activity, outliers were defined as ≥3 standard deviations from the mean and were excluded from the VBM analyses.

### MRI acquisition

3-T data scanning were on collected at the University of Tokyo (Prisma, Siemens, Erlangen, Germany; 43 males and 11 females), Kyoto University (3-T Siemens Verio; 22 males and 38 females), and RIKEN (3-T Siemens Prisma; 15 males and 15 females), with 32-channel head coils. Three-dimensional, high-resolution, T1-weighted structural images were collected using a magnetization-prepared rapid gradient-echo (MPRAGE) sequence. The parameters of each facility were as follows: (University of Tokyo) a 256 × 256 matrix, repetition time (TR) = 1.9 ms, echo time (TE) = 2.53 ms, inversion time (TI) = 900 ms, field of view (FOV) = 24 cm, 192 slices, 1.0 mm slice thickness; (Kyoto University) 240 × 256 matrix, TR = 1.9 ms, TE = 2.52 ms, TI = 900 ms, FOV = 24 cm, 192 slices, 1.0 mm slice thickness; (RIKEN) 256 × 256 matrix, TR = 2.4 ms, TE = 2.19 ms, TI = 1060 ms, FOV = 24 cm, 192 slices, 1.0 mm slice thickness.

### Voxel-based morphometry (VBM)

The brain images were analysed using a MATLAB R2014a (MathWorks Inc., Natick, MA) extension written for Statistical Parametric Mapping 12 (SPM12; Welcome Department of Imaging Neuroscience, London, UK), and the CAT12 toolbox written by Gaser (http://www.neuro.uni-jena.de/cat). A unified segmentation was used, which combined both normalization and segmentation parameters within the same generative model^38^. The output GM, white matter (WM), and cerebrospinal fluid (CSF) image partitions were resliced into 1.5 × 1.5 × 1.5 mm voxels. The voxel values of segmented and normalized GM images were modulated by the Jacobian determinants obtained from a non-linear normalization step. The resultant GM images on which all analyses were performed were smoothed with Gaussian kernels of 8 mm full-width-at-half-maximum.

### Statistical analysis

We compared the daily time and weekly range of each activity on the Life-Log between males and females with two-sample unpaired tests using IBM SPSS Statistics for Windows 23.0 (IBM Corp., Armonk, NY, USA). All variables were examined for normality of distribution using the Shapiro-Wilk test. We analysed measurements that were and were not normally distributed using t-tests and the Mann-Whitney U test, respectively. For each of the 24 activity categories, we assessed daily time and weekly range as well as positive and negative correlations for males and females separately. We excluded three activity items for male (Schoolwork, Nursing/Caring, Hospital Visit/Treatment) and one item for female (Hospital Visit/Treatment) participants because all values were zero with the exception of outliers. As a result, we obtained 176 statistical models (84 for male and 92 for female participants). To adjust for the effects of age and individual differences in head size, we entered age and total intracranial volume (TIV) as nuisance covariates according to previous studies^39^. The TIV was estimated by summing up the voxel-values of tissue for GMV, WM volume, and CSF volume, and was used to control for individual differences in overall head size^40^. The cluster-defining threshold was set to p < 0.001, and a family-wise error-corrected (FWE) cluster size threshold of p < 0.05 was applied to account for multiple comparisons (corrected cluster sizes). Our primary purpose was to comprehensively analyse all of the available variables, and the exploratory nature of our study generated a large number of statistical models. Thus, we reported all the results uncorrected for the number of models examined, despite the possibility of a higher rate of false positives due to multiple comparison problems. Finally, cluster sizes were adjusted for smoothness non-uniformity using the CAT12 toolbox extension for SPM12 by Gaser (http://www.neuro.uni-jena.de/cat/), which implemented the methodology used by Hayasaka *et al*.^41^. The anatomical localisation of significant clusters was performed using the Neuromorphometrics atlas provided by the CAT12 toolbox. To further examine sex-specificity, we examined sex by activity-measure interactions in GMV for each of the 11 clusters in which significant correlations were found. As with the correlation analysis, the effect of age and TIV were excluded from the data as nuisance covariates. Activity variables (daily time or weekly range) were split into two columns of covariates by sex.

### Data Availability

The datasets generated and analysed during the current study are available from the corresponding author upon reasonable request.

## Electronic supplementary material

Supplementary Information
