# Supplementary material for: Sex-specific regional grey matter volume correlates of daily activities
Source: Sci Rep. 2018 Jul 2;8:9935. doi: 10.1038/s41598-018-28252-w (PMC6028590; doi:10.1038/s41598-018-28252-w)

Sex-specific regional grey matter volume correlates of daily activities

Tsukasa Ueno^1^, Naoya Oishi^2*^, Toshiya Murai^1^

**^*^Corresponding author**

Dr Naoya Oishi

Research and Educational Unit of Leaders for Integrated Medical System

Center for the Promotion of Interdisciplinary Education and Research

Kyoto University

54 Kawahara-cho, Shogoin, Sakyo-ku, Kyoto 606-8507, Japan

Tel: +81-75-751-3695; Fax: +81-75-751-3202; E-mail: [noishi@kuhp.kyoto-u.ac.jp](mailto:noishi@kuhp.kyoto-u.ac.jp)

Supplementary Table S1. Physical characteristics of the participants.

|  | Male (n = 80) | | | Female (n = 64) | | |
| --- | --- | --- | --- | --- | --- | --- |
|  | Mean | SD | Range | Mean | SD | Range |
| Age (years) | 49.7 | 8.1 | 25–69 | 46.9 | 7.7 | 32–68 |
| Body Mass Index (BMI) | 22.9 | 3.2 | 17.6–34.3 | 21.1 | 3.5 | 14.6–34.1 |
| Systolic Blood Pressure (mmHg) | 127.7 | 14.8 | 97–167 | 120.7 | 18.1 | 83–167 |
| Diastolic Blood Pressure (mmHg) | 82.9 | 10.8 | 63–110 | 74.2 | 11.8 | 46–105 |
| Heart Rate (per minute) | 70.5 | 10.4 | 49–101 | 71.5 | 11.4 | 49–105 |

Supplementary Table S2. Descriptive statistics of the participants.

|  | Male (n = 80) | | | Female (n = 64) | | |
| --- | --- | --- | --- | --- | --- | --- |
| Daily activities (hours) | Mean | SD | Range | Mean | SD | Range |
| High Activity Days |  | | | | | |
| Number of days spent for high activity | 4.5 | 1.0 | 1.0–6.0 | 2.1 | 1.2 | 1.0–7.0 |
| Sleep | 6.3 | 0.9 | 4.0–9.0 | 7.4 | 1.3 | 3.0–9.0 |
| Personal Care | 1.0 | 0.7 | 0.0–3.7 | 1.1 | 0.9 | 0.0–7.5 |
| Meals | 1.6 | 0.6 | 0.0–4.0 | 2.0 | 0.9 | 0.0–3.0 |
| Commuting to Work/School | 2.2 | 0.9 | 0.0–4.5 | 0.0 | 0.3 | 0.0–10.7 |
| Job-work | 9.4 | 2.5 | 0.0–14.0 | 0.2 | 1.6 | 0.0–11.2 |
| School-work | 0.0 | 0.0 | 0.0–0.0 | 0.0 | 0.2 | 0.0–6.2 |
| Domestic-work | 0.1 | 0.4 | 0.0–3.5 | 3.4 | 2.6 | 0.0–12.5 |
| Caring or Nursing | 0.0 | 0.0 | 0.0–0.5 | 0.0 | 0.2 | 0.0–2.5 |
| Child Care | 0.0 | 0.2 | 0.0–1.7 | 0.4 | 2.1 | 0.0–5.0 |
| Shopping | 0.1 | 0.4 | 0.0–3.0 | 0.9 | 1.1 | 0.0–6.5 |
| Non-commute Travel | 0.2 | 0.8 | 0.0–7.0 | 0.5 | 1.0 | 0.0–5.0 |
| Television/Radio/Newspaper/Magazine | 1.1 | 1.3 | 0.0–8.5 | 2.6 | 2.7 | 0.0–8.5 |
| Rest/Relaxation | 0.9 | 1.0 | 0.0–5.0 | 2.3 | 2.3 | 0.0–5.5 |
| Learning/Self-development | 0.1 | 0.4 | 0.0–2.5 | 0.2 | 0.8 | 0.0–3.0 |
| Hobbies/Amusements | 0.1 | 0.5 | 0.0–3.0 | 1.0 | 2.1 | 0.0–3.7 |
| Sports | 0.1 | 0.8 | 0.0–7.7 | 0.2 | 0.7 | 0.0–7.5 |
| Volunteer/Community Activity | 0.0 | 0.4 | 0.0–3.5 | 0.2 | 1.3 | 0.0–1.5 |
| Other Social Activities | 0.2 | 0.7 | 0.0–4.0 | 0.7 | 1.9 | 0.0–3.0 |
| Hospital Visit/Treatment | 0.0 | 0.1 | 0.0–1.0 | 0.0 | 0.0 | 0.0–0.7 |
| Other Activities | 0.0 | 0.0 | 0.0–0.5 | 0.0 | 0.0 | 0.0–4.0 |
| Being Alone | 7.6 | 4.9 | 0.0–24.0 | 6.8 | 7.7 | 0.0–19.2 |
| Being with Family | 5.6 | 4.3 | 0.0–16.5 | 11.2 | 9.0 | 0.0–24.0 |
| Being with Classmates/Colleagues | 9.3 | 4.0 | 0.0–15.0 | 0.1 | 1.0 | 0.0–13.5 |
| Being with Other People | 0.8 | 2.0 | 0.0–9.0 | 1.2 | 2.5 | 0.0–4.0 |
| Low Activity Days (hours) |  | | | | | |
| Number of days spent for low activity | 1.8 | 0.6 | 1.0–5.0 | 2.1 | 1.2 | 4.5–14.0 |
| Sleep | 7.7 | 1.4 | 4.0–11.0 | 7.4 | 1.3 | 0.0–5.0 |
| Personal Care | 1.4 | 1.1 | 0.0–5.0 | 1.1 | 0.9 | 0.0–4.5 |
| Meals | 2.1 | 0.9 | 0.0–5.0 | 2.0 | 0.9 | 0.0–2.0 |
| Commuting to Work/School | 0.1 | 0.4 | 0.0–3.0 | 0.0 | 0.3 | 0.0–11.0 |
| Job-work | 0.4 | 1.8 | 0.0–12.0 | 0.2 | 1.6 | 0.0–2.0 |
| School-work | 0.0 | 0.2 | 0.0–2.0 | 0.0 | 0.2 | 0.0–11.0 |
| Domestic-work | 0.6 | 0.9 | 0.0–3.5 | 3.4 | 2.6 | 0.0–2.0 |
| Caring or Nursing | 0.0 | 0.2 | 0.0–2.2 | 0.0 | 0.2 | 0.0–16.0 |
| Child Care | 0.4 | 1.4 | 0.0–7.5 | 0.4 | 2.1 | 0.0–5.0 |
| Shopping | 0.9 | 1.0 | 0.0–3.0 | 0.9 | 1.1 | 0.0–4.5 |
| Non-commute Travel | 0.5 | 0.9 | 0.0–3.2 | 0.5 | 1.0 | 0.0–11.2 |
| Television/Radio/Newspaper/Magazine | 2.9 | 2.4 | 0.0–10.5 | 2.6 | 2.7 | 0.0–14.7 |
| Rest/Relaxation | 2.5 | 2.1 | 0.0–7.0 | 2.3 | 2.3 | 0.0–4.7 |
| Learning/Self-development | 0.2 | 0.9 | 0.0–6.2 | 0.2 | 0.8 | 0.0–11.5 |
| Hobbies/Amusements | 1.9 | 2.4 | 0.0–10.5 | 1.0 | 2.1 | 0.0–4.0 |
| Sports | 1.1 | 1.8 | 0.0–10.7 | 0.2 | 0.7 | 0.0–9.7 |
| Volunteer/Community Activity | 0.1 | 1.3 | 0.0–12.0 | 0.2 | 1.3 | 0.0–10.0 |
| Other Social Activities | 0.3 | 1.4 | 0.0–11.5 | 0.7 | 1.9 | 0.0–0.0 |
| Hospital Visit/Treatment | 0.0 | 0.0 | 0.0–0.0 | 0.0 | 0.0 | 0.0–0.5 |
| Other Activities | 0.1 | 0.5 | 0.0–4.5 | 0.0 | 0.0 | 0.0–24.0 |
| Being Alone | 9.4 | 7.4 | 0.0–24.0 | 6.8 | 7.7 | 0.0–24.0 |
| Being with Family | 12.0 | 7.9 | 0.0–24.0 | 11.2 | 9.0 | 0.0–8.0 |
| Being with Classmates/Colleagues | 0.2 | 1.5 | 0.0–10.0 | 0.1 | 1.0 | 0.0–11.0 |
| Being with Other People | 1.1 | 2.6 | 0.0–13.0 | 1.2 | 2.5 | 3.0–14.0 |

Supplementary Figure S1. A representative case of a 24-hour Life-Log on a High Activity day.

Q1. Please indicate the number of High activity days in a week. Note that the sum of high activity and low activity days should be 7.

→ 5 days

Q2. Please record your representative activities for an average High Activity day in the past year, such as a weekday, by filling out each 15-minute cell with a horizontal line. Also, please draw a horizontal line to specify who was with you on an average High Activity day for each 15-minute period.

Note that more than two activities should not be recorded for any 15-minute cell.


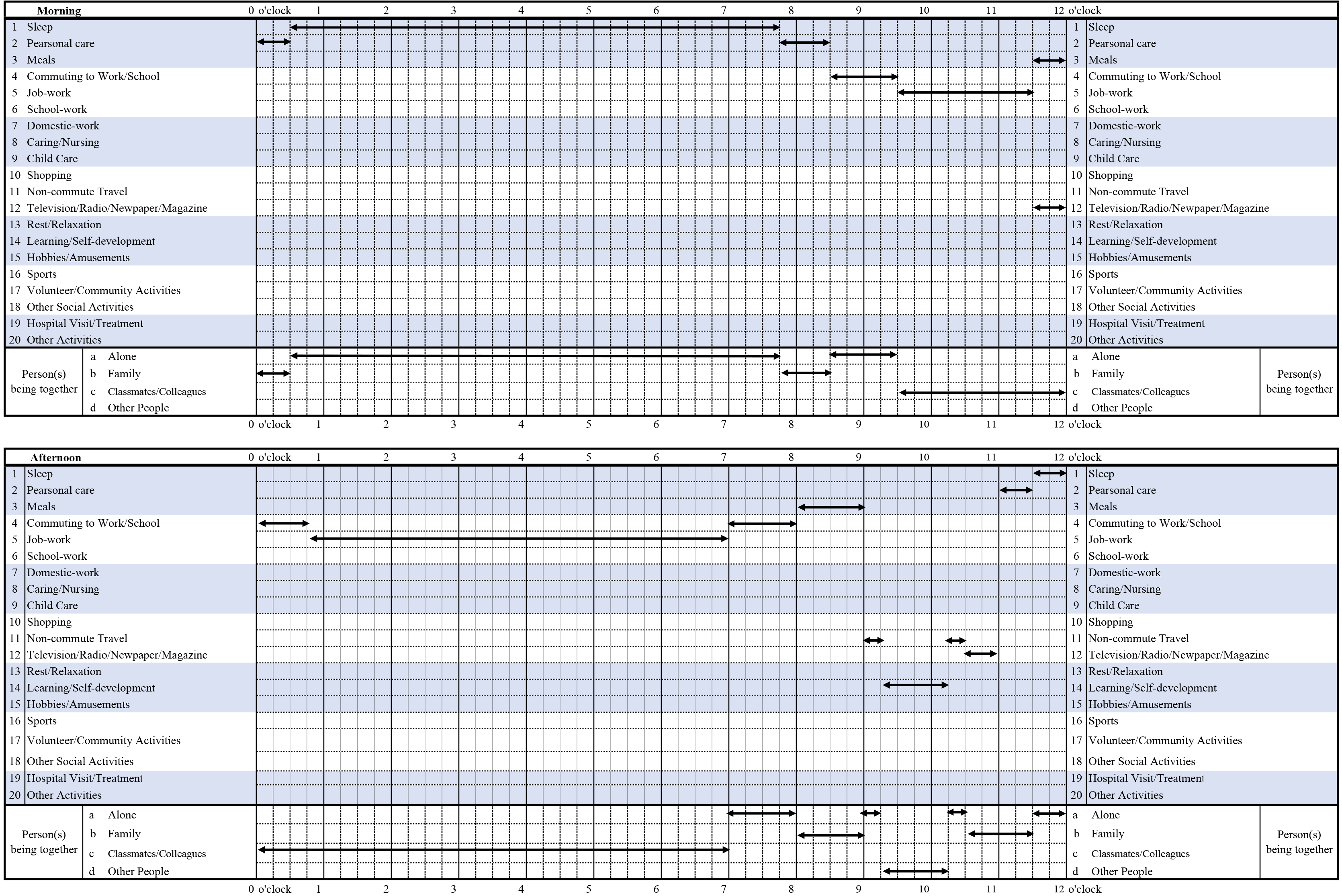


Supplementary Figure S2. A representative case of 24-hour Life-Log on a Low Activity day.

Q1. Please indicate the number of Low activity days in week. Note that the sum of high activity and low activity days should be 7.

→ 2 days

Q2. Please record your representative activities for an average Low Activity day in the past year, such as a day-off, by filling out each 15-minute cell with a horizontal line. Also, please draw a horizontal line to specify who was with you on an average Low Activity day for each 15-minute period.

Note that more than two activities should not be recorded for any 15-minute cell.


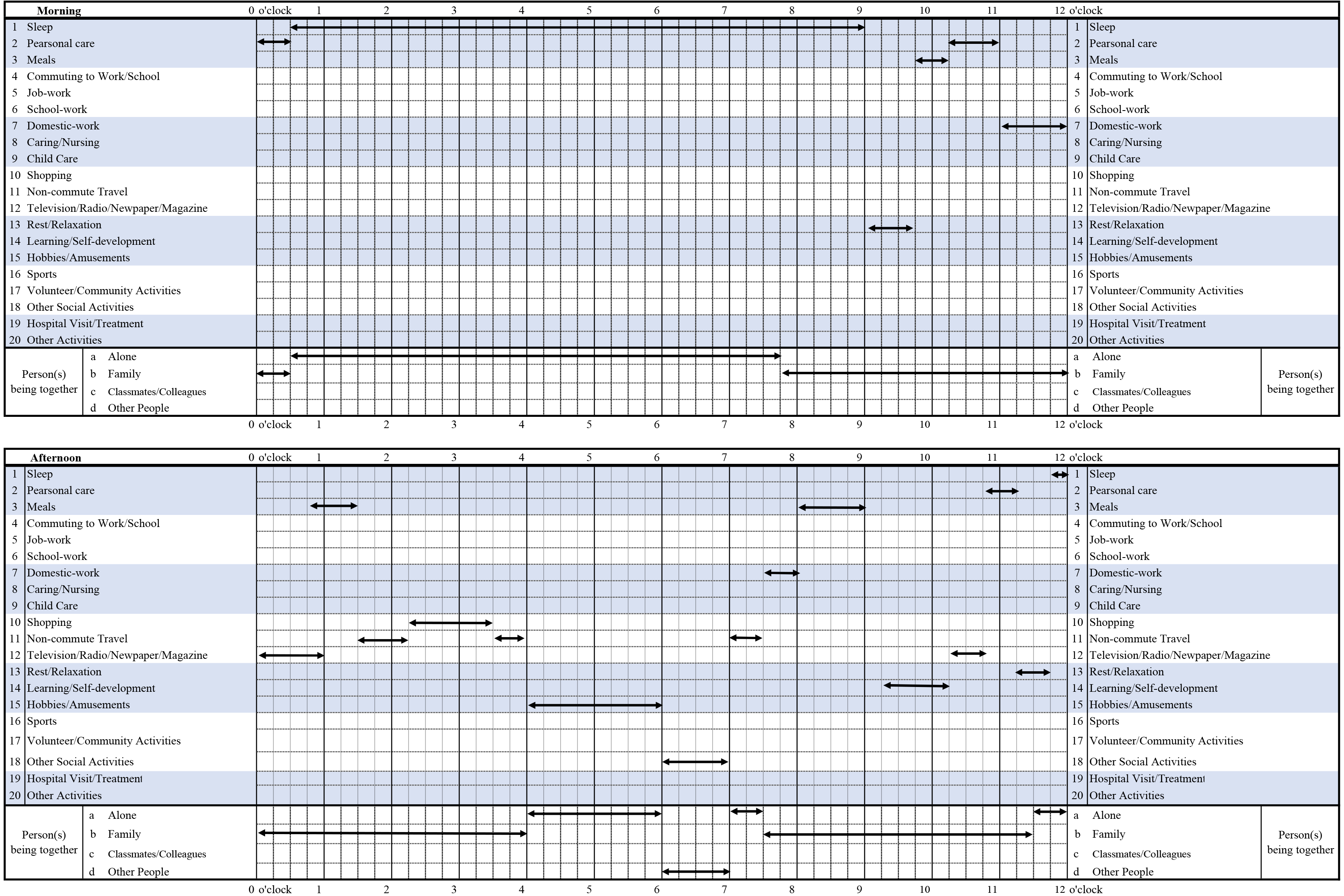

Supplement: Supplementary file 1 — Supplementary Information [file 41598_2018_28252_MOESM1_ESM.docx]
